# PALLIHIP-study: Spinal Phenol IN Glycerol (SPING-block) in palliative, frail, older adults with a hip fracture: a study protocol

**DOI:** 10.1186/s12877-026-07377-y

**Published:** 2026-03-23

**Authors:** Nick Visschers, Miriam van der Velden, Miriam C. Faes, Bart Spaetgens, Marieke van den Beuken-van Everdingen, Arnela Suman

**Affiliations:** 1 Department of Pain Medicine and Palliative Care, Amphia Hospital, Breda, The Netherlands; 2 Department of Geriatrics, Amphia Hospital, Breda, The Netherlands; 3 Department of Internal Medicine, Geriatrics Section, Maastricht University Medical Centre+, Maastricht, The Netherlands; 4 Care and Publich Health Research Institute (CAPHRI), Maastricht University, Maastricht, The Netherlands; 5 Centre of Expertise in Palliative Care, Maastricht University Medical Centre, Maastricht, The Netherlands

**Keywords:** Femur fracture, Frail, older individuals, Palliative care, Pain treatment, SPING block, Conservative treatment, Nonoperative management

## Abstract

**Background:**

In the Netherlands, approximately 20,000 older adults sustain a hip fracture every year. Most of these patients are frail, institutionalised, have limited postoperative mobility and a high mortality rate. When surgery is not feasible or preferred, optimizing quality of life and effective pain management becomes paramount. However, current analgesic strategies often fail to provide sustained relief and timely discharge. Timely discharge is essential to prevent hospital-related complications in frail patients. The Spinal Phenol IN Glycerol (SPING) block, a novel intrathecal neurolytic technique adapted from spinal anaesthesia, has been developed as an innovative approach for palliative pain treatment in this patient population. The PALLIHIP study aims to evaluate the effects of the SPING block on pain and quality of life in frail, institutionalised older adults with hip fractures.

**Methods:**

PALLIHIP is a prospective multicentre cohort study that will be conducted in 19 Dutch hospitals. PALLIHIP will aim to enroll 210 patients. The primary objective is to evaluate the impact on pain and quality of life, assessed at admission, 24 h, one week, six weeks, and three months after treatment with SPING. Secondary outcomes include complications, mobility level, care dependency, caregiver burden, quality of dying, survival, and patient and caregiver satisfaction. Patient characteristics associated with treatment success will be explored. Healthcare and societal costs will be estimated through activity-based costing and a budget impact model. Semi-structured interviews and focus groups with patients, informal caregivers, and healthcare professionals will explore experiences, preferences, and implementation barriers. Ultimately, the goal is to establish a framework with a standardised protocol for effective, consistent application of the SPING block in clinical practice.

**Discussion:**

This study will deliver the first prospective data on clinical effectiveness, patient-centred outcomes, and economic impact of the SPING block in frail, older adults with hip fractures. We hypothesise that the SPING block provides rapid, sustained analgesia with reduced opioid requirements, facilitates early discharge, and improves patient-centered outcomes. Findings may be able to provide evidence to inform future national guideline development and support the development of a standardised protocol for nonoperative palliative fracture care in frail, institutionalised older adults with a hip fracture.

**Trial registration:**

Clinical trial number: not applicable. The PALLIHIP study is an observational cohort study on palliative fracture care in frail older adults with a hip fracture and was deemed exempt from the Medical Research Involving Human Subjects Act (WMO) by the Medical Ethics Committee of Amphia Hospital (protocol number NW2024-049).

## Background

Approximately 20,000 patients with hip fractures are admitted to hospitals across the Netherlands annually [1]. These patients are mostly frail, older adults, of whom 20% live in nursing homes [2]. Frailty among older adults is characterized by an accumulation of limitations in physical, psychological, and social functioning [3]. Frailty is a strong predictor of mortality and poor functional outcomes after surgery [3, 4]. In this patient population, the overall one-year mortality rate after surgery is 25%. This is mainly due to advanced age, multiple comorbidities, cognitive impairments, and limited mobility [5]. Following surgery, only 10–20% of these patients can walk a few steps or a short distance [5, 6].

Therefore, surgical treatment as the gold standard for hip fractures in frail, older adults is questionable. Non-operative management (NOM) is considered when surgery is not feasible (due to e.g., severe frailty, advanced dementia, multimorbidity, limited pre-fracture mobility, or high preoperative risk) or not preferred (e.g., when expected benefits of surgery do not align with the patient’s values, comfort goals, or anticipated quality of life). In 2023, 4.8% of Dutch patients presenting at emergency departments with a hip fracture did not receive surgery, and this number is increasing [7]. Providing adequate pain relief, especially during movement and care, and a timely hospital discharge are key objectives when opting for a non-operative approach. Timely hospital discharge is essential in this frail population as prolonged hospitalisation increases the risk of delirium, infections, functional decline, and distress caused by separation from their familiar environment and caregivers [8, 9].

A substantial proportion of frail, institutionalised, older adults for whom non‑operative management is considered are already in, or approaching, a palliative phase of care. In this context, treatment goals shift from life‑prolongation to optimising comfort, minimising treatment burden, and supporting personalised end‑of‑life priorities. However, current pain management options within palliative non‑operative pathways remain limited and often insufficient to meet these goals. Treatment options mainly involve opioids, which are associated with several side effects, such as sedation, increased cognitive dysfunction, respiratory depression, and nausea in frail, older adults [4]. Current locoregional techniques do not guarantee long-term pain reduction, high success rates, or rapid discharge. For example, the fascia iliaca compartment block and the femoral nerve block are typically administered as a single-shot local anaesthesia or through an in-situ catheter [10, 11]. These methods are not suitable for therapy within primary care or for long-term use. The pericapsular nerve block (PENG) using phenol or alcohol may not provide adequate pain relief for subtrochanteric fractures and does not directly enable sitting upright without opioids. Furthermore, PENG requires significant practitioner expertise, resulting in a steep learning curve that limits its suitability and accessibility [12, 13].

To address these challenges, the Spinal Phenol IN Glycerol (SPING) block has been developed as an innovative, palliative pain treatment for frail, older adults with a hip fracture [14]. This technique is adapted from spinal anaesthesia as a technique where anaesthetics are administered directly into the intrathecal space. These intrathecal injections are a routine daily practice for anaesthesiologists in the Netherlands. The modified technique, where phenol is used as a neurolytic agent, has been previously described in literature as a promising treatment for pain with spasms [15–17], for treatment in oncological pain [18], and for hip fractures in two case reports [19, 20]. The recent further advancement of the technique and its application in frail, older adults as a non-operative treatment for hip fractures was developed in 2021 in the Amphia hospital, and preliminary results have been described in a case-series paper published in the Dutch Journal of Medicine (Nederlands Tijdschrift voor Geneeskunde) [14]. This paper demonstrated that the SPING block was successfully administered and reached almost immediate pain relief in ten patients, regardless of the type of hip fracture. Patients were discharged timely, with a mean hospital stay of 1.9 days (SD 0.9), and showed high satisfaction with the SPING block treatment and the acquired pain relief. Patients treated with SPING block maintained adequate pain relief without requiring any additional opioid analgesia, throughout the routine follow-up after a pain intervention. Most of these patients (80%) could be released to their previous living environment.

Factors such as adequate pain management and proximity to loved ones are key determinants of patient comfort and overall quality of life (QoL) [2]. Given that pain, immobility, and treatment‑related adverse effects are major contributors to reduced QoL in frail older adults with hip fractures [6], an intervention that provides rapid analgesia, minimises opioid requirements, enables early upright sitting and transfers, and potentially shortens hospital stay may meaningfully enhance patient well‑being.

The capacity of SPING to deliver fast and sustained analgesia, reduce opioid requirements, and support early mobilisation therefore suggests that SPING may offer a more substantial QoL benefit for frail older adults compared with existing non‑operative treatment options. These potential advantages must be carefully balanced against the associated disadvantages of the technique, including irreversible motor loss, potential neuropathic complications, and a risk of faecal or urinary incontinence.

While the SPING block is increasingly showing its clinical value, further research is needed to fully establish its effects on sustained analgesia, functional outcomes, and overall QoL in frail older adults and to integrate it into non-operative care protocols consistently.

## Methods/design

### Study design and setting

PALLIHIP is a prospective, observational, multicentre cohort study that will be conducted in 19 Dutch hospitals without random assignment. Patient characteristics associated with treatment success will be explored, such as baseline frailty, cognitive impairment severity, pre-fracture mobility level, comorbidities, and pain severity at admission. Healthcare and societal costs will be estimated through activity-based costing and a budget impact model. Semi-structured interviews and focus groups with patients, informal caregivers, and healthcare professionals (e.g., physicians, physician assistants, nurse practitioners, and other qualified clinicians) will explore experiences, preferences, barriers, and facilitators for implementation of the SPING block in routine non-operative treatment of hip fractures. Participating centres include: Amphia Hospital, Bernhoven Hospital, Diakonessenhuis, Franciscus Hospital, Frisius Medical Centre, Groene Hart Hospital, Haaglanden Medical Centre, Haga Hospital, Ikazia Hospital, Isala Hospital, Maasstad Hospital, Nij Smellinghe Hospital, Rijnstate Hospital, Rivierenland Hospital, Slingeland Hospital, Spaarne Gasthuis, St. Antonius Hospital, Treant Hospital Group, Zuyderland Medical Centre.

### Objectives and work packages (WP)

The PALLIHIP study will be designed with the following objectives: (1) The primary objective is to describe the impact of the SPING block on pain and QoL in frail, older adults with a hip fracture, who have received the SPING block treatment; (2) To identify patient characteristics that may predict a successful treatment outcome after treatment with the SPING block; (3) To analyse the economic costs and benefits of the SPING block for this group of patients, and to model the budget impact from both a healthcare and societal perspective in preparation for widespread adoption of the SPING block as standard care; (4) To provide insight into the experiences, preferences and needs of frail, older adults with a hip fracture and their informal caregivers, during the process of decision making regarding the treatment of the hip fracture and the choice for SPING block; (5) To develop a standardised treatment protocol for the SPING block as non-operative treatment for frail, older adults with a hip fracture in the palliative phase.

To address the identified knowledge gaps and problem definition, PALLIHIP has the following objectives, which will be addressed in various work packages (WP). The quantitative component (Work packages 1 and 2) will prospectively include all frail patients receiving the SPING block over 36 months. The qualitative component (Work packages 3 and 4) will use semi-structured interviews and focus groups with patients, informal caregivers, and anesthesiologists/palliative care clinicians, as well as geriatricians and nursing home physicians (specialists in long-term and nursing home medicine, unique to the Dutch healthcare system [21]). and orthopedic/trauma surgeons to investigate decision-making and barriers and facilitators for implementation of the SPING block. Ultimately, the goal is to establish a standardised framework and protocol for effective and consistent application of the SPING block in clinical practice.

### WP 1: Impact of SPING block and identification of patient characteristics

#### Primary research question

What proportion of patients with a hip fracture who are treated with the SPING block have a successful treatment outcome?

#### Objective

To describe the impact of the SPING block on pain and QoL in frail, older adults with a hip fracture, who will opt for SPING block treatment as NOM for their hip fracture. To identify patient characteristics that are associated with a successful treatment outcome after treatment.

#### Methods

A prospective, observational, multicentre cohort study to describe the impact of the SPING block on various outcomes reported by both patients, informal and professional caregivers, in the hospital and nursing home.

### WP 2: Costs and benefits of the SPING block

#### Research question

What are the financial consequences of national implementation of the SPING block?

#### Objective

To analyse the economic costs and benefits of the SPING block for this patient population, and to model the budget impact from both a healthcare and societal perspective in preparation for widespread implementation of the SPING block.

#### Methods

A cost analysis using an activity‑based costing approach will be conducted to estimate the direct costs of the SPING block. Activities involved in SPING block (e.g.: 30 min in the recovery room, sterile set for epidural, admission to care department for on average 1.1 days) will be captured as part of the observational study, using administrative data and hospital records. To evaluate the broader financial implications of national adoption, a decision‑analytic model will be developed. This model will estimate the budget impact at one, two, and three years after implementation. Literature, registration data, hospital data, and expert opinion will inform several assumptions in the model, such as the expected patient population (incidence) per year, expected costs of standard care, and level of substitution (adoption) of the SPING block. The base-case analysis of the budget impact will be from a healthcare perspective, focusing only on hospital and medication costs. If possible, a scenario analysis will be conducted that includes changes in healthcare utilization due to changes in outcomes (e.g. side-effects and/or adverse events).

### WP 3: Patients and their informal caregivers

#### Research question

How do patients with a hip fracture and their informal caregivers experience shared decision-making regarding SPING block treatment in a palliative, non-operative care setting?

#### Objective

To provide insight into the experiences, preferences, and needs of frail older adults with a hip fracture and their informal caregivers regarding the decision-making process for hip fracture treatment and the choice of SPING block treatment.

#### Methods

A qualitative study using semi-structured interviews. Purposive sampling, which involves a deliberate selection of study participants, will be applied. The interview topic list will be drawn from prior key literature, including findings from the FRAIL HIP and PENG studies [22–24], as well as the experiences of informal caregivers and patients. The results of this study will be used to develop information materials for patients and informal caregivers, and to inform the development of a standardised treatment protocol (WP 4).

### WP 4: Standardised treatment protocol

#### Research question

How do healthcare professionals experience the use of SPING in palliative care for hip fractures, and what needs do they identify in current practice?

#### Objective

To develop a standardised treatment protocol for the SPING block as a non-operative treatment for frail, older adults with a hip fracture in the palliative phase, based on experiences, needs, and contextual factors identified by healthcare professionals.

#### Methods

A qualitative study using semi-structured interviews and focus groups with healthcare professionals involved in the care of frail, older adults with hip fractures. The study explores their experiences and needs with applying the SPING block in clinical practice, their perceived needs and expectations, and contextual barriers and facilitators that influence acceptance and application of the SPING block treatment.

### Study population and eligibility criteria

Distinct participant populations and recruitment strategies have been defined per work package to align with their respective methodological approaches.

### Patients (WP 1 and WP 2)

Frail, institutionalized adults admitted with a hip fracture who opt for SPING block as their non-operative pain management will be enrolled. Inclusion **(**all criteria must apply): Age ≥ 70 years, acute unilateral post-traumatic hip fracture, confirmed by radiography, clinical Frailty Scale > 6, American Society of Anaesthesiologists class > 3, functional ambulation categories ≥ 2, and opting for SPING block in shared decision-making. Exclusion: SPING block for any other indication than a hip fracture, such as oncological indication, uncontrolled dementia wandering, or inability/unwillingness to give informed consent.

### Informal caregivers (WP 3)

The primary informal caregiver is defined as the non-professional who is most involved in caring for the older patient with a hip fracture, assists with at least one personal or instrumental activity of daily living, and monitors the patient. Inclusion criteria (all criteria must apply): Primary informal caregiver of an enrolled patient, actively engaging in shared decision-making (SDM), and opting for SPING block as part of their non-operative treatment. Exclusion: Patient survival ≤ 7 days after SPING block to avoid interference with the acute grief processes or inability/unwillingness of the informal caregiver to give informed consent.

### Healthcare professionals (WP 4)

Healthcare professionals at participating sites who perform or directly support SPING block procedures in the context of palliative care for patients with a hip fracture. Inclusion criteria **(**all criteria must apply): Employment at a clinical site participating in the PALLIHIP study, have directly performed or supported SPING block procedures for a patient with a hip fracture, and are involved in clinical decision-making regarding pain management and/or palliative care for frail older adults. Exclusion: No direct clinical experience with SPING block procedure or inability/unwillingness to give informed consent.

### Recruitment procedures

#### Patients

At admission to the emergency department, the attending surgical resident will evaluate the patient. According to standard hospital protocol, the geriatrician will be consulted in case of a hip fracture in frail, older adults. If the patient is frail and their informal caregivers and/or physicians doubt whether surgical treatment is the most appropriate option, an SDM process will commence within 24 h. This SDM process involves the patient and/or the informal caregiver, an orthopaedic surgeon, a geriatrician, and an anaesthesiologist specialised in invasive pain treatment and palliative care and follows the different steps of the SDM model of Elwyn et al. (25).

During this process, the benefits of surgical treatment and the estimated probability of return to a meaningful level of mobility will be weighed against the patients’ needs and values and perioperative risks. Subsequently, the advantages of both surgical and NOM will be discussed with the patient and/or their informal caregiver. For those opting for non-operative treatment, the anaesthesiologist/pain specialist will discuss the pain management options, including ongoing medication strategies, such as paracetamol and opioids, according to the pain protocol as set at admission, or the SPING block. If the SPING block is chosen, detailed treatment information will be provided both orally and in writing (information brochure), and treatment consent will be obtained by the anaesthesiologist/pain specialist or research associate. Subsequently, the patient and/or the informal caregiver are informed about the PALIHIP study by the treating anesthesiologists or research associate. They will receive written study information, after which written consent will be obtained. Throughout the recruitment process, participants will be made aware that their involvement in the research is voluntary, and they may withdraw at any time without consequences. The SPING block procedure is performed within 24 h after the SDM discussion, and patients will usually be transferred back to the nursing department within 30 min post-procedure, with hospital discharge following as soon as clinically feasible.

### Informal caregivers

Caregivers will be selected using purposive sampling to ensure a diverse range of experiences with the SPING block and contacted by the research team for study participation. Caregivers of patients who survived less than seven days following treatment will be excluded to avoid potential interference with the acute grieving process. Verbal consent will be obtained initially, and written consent will be collected before any audio recordings for the interviews are made. They will be informed that they may withdraw from the study at any time without consequences.

#### Professionals

Site investigators will contact all anesthesiologists, pain physicians, nurse anaesthetists, and palliative care specialists who have directly performed or supported SPING block procedures for a patient with a hip fracture using the preliminary protocol. Those who respond will be screened for eligibility and sent a study information sheet and consent form. At the start of each interview or focus group, verbal consent to record the session will be confirmed, followed by signing the written consent. All professionals will be reminded that participation is voluntary and that they may withdraw at any time.

### Outcomes and measurements

#### Primary outcome

The primary outcomes are improvements in pain scores, measured using the Numeric Rating Scale (NRS) or the Pain Assessment Checklist for Seniors with Limited Ability to Communicate – Dutch (PACSLAC-D), and health-related quality of life (EQ-5D-5 L). A successful outcome is defined as a reduction in pain to a score of 1–3 during nursing activities within 24 h after SPING block without the need for additional analgesics and sustained until death or for at least three months after SPING block.

### Secondary outcomes

Changes in pain scores over time (pain trajectories) at rest and during movement (NRS or PACSLAC-D), cumulative opioid consumption (Oral morphine equivalents), SPING-related adverse events (E.g. urine incontinence/retention, fecal incontinence/obstipation, delirium, bleeding, infection, hypotension, neuropathic pain), functional mobility status (bedbound, bed-chair transfer, able to walk a few steps (< 10 m), mobile (> 10 m)), level of care dependency (CDS), patient and/or informal caregiver satisfaction with chosen treatment, perceived quality of dying and death (QODD), time to hospital discharge and date of death, healthcare resource and costs (iMTA Medical Consumption Questionnaire) and stakeholder experiences and barriers and facilitators for implementation (qualitative thematic analysis). The secondary outcomes will be assessed at the same measurement time points as the primary outcomes (admission, within 24 h after treatment, one week, six weeks, and three months after treatment). The outcome measurements are described in Table 1, outcome measures.

**Table 1:** Outcome measures

Outcome measure	Measurement instrument	At hospital admission	Within 24 h after treatment	One week after treatment	Six weeks after treatment	Three months after treatment
Pain score during nursing activities	NRS or PACSLAC-D (26)	x	x	x	x	x
Pain score in rest	NRS or PACSLAC-D (26)	x	x	x	x	x
Quality of life	EuroQoL-5D (EQ-5D-L) (27)		x	x	x	x
Complications	Paresis of the affected leg, urine incontinence/retention, faecal incontinence/obstipation, delirium, bleeding, infection, hypotension, other		x	x	x	x
Additional analgesics	Yes or no, morphine equivalent		x	x	x	x
Mobility level	Bedbound, bed-chair transfer, able to walk a few steps (< 10 m), mobile (> 10 m)	x	x	x	x	x
Level of care dependency	Care dependency scale (CDS) (28)		x	x	x	x
Burden on informal caregiver	Zarit Burden Interview Short Form (25)	x			x	x
Satisfaction of the patient (or informal caregiver) with the chosen treatment	11-point Numeric Rating Score. For patients who have deceased, their informal caregiver will be asked to complete this.					x
Survival rate	Patient deceased or not, if so: date of death			x	x	x
Quality of dying and death	Quality of dying and death interview QODD (30)				x	x

### Questionnaires

Questionnaires will be administered to patients where possible, or to (informal) caregivers in cases where a patient is unable to respond for themselves. The following validated instruments will be used for outcome assessment: Numerical Rating Scale (NRS) and the Pain Assessment Checklist for Seniors with Severe Dementia (PACSLAC-D) [26], EurQol-5-Dimension-5-Level (EQ-5D-5 L) for QoL [27], for pain, CDS for the Level of care dependency [28], and Zarit Burden Interview for caregiver burden [25], and Quality of Dying and Death Questionnaire (QODD) for quality of dying and death [29].

### Data management and monitoring

All required data for PALLIHIP will be sourced primarily from the electronic patient record (EPD). Variables not routinely captured in the EPD will be collected additionally and stored in encrypted form on each hospital’s secure server. All data will be pseudonymised. The key file containing this link is accessible per centre only to the executive researcher and the relevant principal investigator (PI), under the PI’s responsibility. Pseudonymised datasets will be exported to Castor EDC (ISO 9001, ISO 27001, NEN 7510). Access to this platform is restricted to PALLIHIP project team members on a need-to-know basis, and an audit trail will be implemented in the platform. Requests for secondary use of the data will be reviewed by the project lead before approval. Data quality will be ensured by systematically identifying extreme and missing values. Any deviations will be addressed directly with the local research team, and efforts will be made to retrieve or verify missing information. PALLIHIP complies fully with Good Clinical Practice guidelines and with the General Data Protection Regulation (GDPR), including the Dutch Implementation Act (UAVG). The FAIR principles (Findable, Accessible, Interoperable, Reusable) are applied throughout. All metadata and datasets will be stored in secure, publicly traceable repositories under clear usage licenses. The project maintains a close collaboration with the Dutch Frail Hip Audit (DFHA). Data exchange and opportunities for reuse between PALLIHIP and DFHA will be evaluated continuously to optimise alignment and efficiency.

### Sample size calculation

We hypothesise that at least 85% of the patients with a hip fracture treated with the SPING block will have a successful treatment outcome. Sample size calculation is based on a two-sided one-sample test for proportion. The significance level is set at an alpha of 0.05 and a power of 0.8. Including approximately 210 patients would lead to a precision (95% confidence interval) of 7% around the effect estimate for this study. The sample size calculation was performed using R version 4.3.1.

### Data analysis / statistical analysis

Descriptive analyses will be performed, reporting proportions/percentages for categorical outcomes, mean (SD) for normally distributed continuous outcomes, and median (IQR) for non-normally distributed continuous outcomes, including 95% CI around the effect estimate. Survival data will be presented using percentages at four weeks, six weeks, and three months, and visually presented using a Kaplan-Meier plot. Prognostic factors for successful treatment outcome will be assessed using logistic regression to estimate Odds Ratios (ORs) with corresponding 95% CI. Missing data will be handled as deemed appropriate according to the underlying missing data pattern.

## Discussion

PALLIHIP will be the first prospective, multicentre study to systematically evaluate the SPING block as a non-operative, palliative treatment strategy for frail, older adults with hip fractures. We hypothesise that the SPING block will fundamentally change the standard of palliative non-operative hip fracture care by delivering rapid and sustained analgesia, reducing opioid exposure, enabling early upright positioning, and facilitating timely discharge, thereby redefining what high-quality comfort-focused care can look like. PALLIHIP addresses an important knowledge gap by examining the impact of the SPING block on pain control, health-related quality of life, and healthcare resource utilization.

In addition to measuring treatment outcomes, PALLIHIP will identify baseline patient characteristics associated with favourable responses to the SPING block, supporting more targeted clinical decision-making. By integrating clinical, economic, and qualitative data (including insights from patients, informal caregivers, and healthcare professionals), the study moves beyond procedural evaluation and aims to inform the development of a standardised national treatment protocol for palliative non-operative hip fracture care.

Beyond evaluating a single intervention, PALLIHIP positions the SPING block within a broader shift in orthogeriatric palliative care. In routine NOM, analgesia often relies on systemic opioids or short-acting peripheral nerve blocks that are not designed to provide sustained comfort in patients with limited life expectancy. Although techniques such as fascia iliaca block, femoral nerve block, and the PENG block may offer short-term analgesia, robust prospective evidence for sustained strategies within palliative NOM trajectories remains limited. The PENG block may offer improved anterior hip analgesia compared to traditional techniques [13]. However, its duration and suitability for end-of-life trajectories remain limited [12, 13]. This underscores the need for more durable neuraxial intervention as NOM. SPING differs mechanistically by delivering intrathecal neurolysis at the spinal level and is specifically developed for patients in whom comfort, and minimising treatment burden are primary goals. This positions SPING as a potentially foundational component of a structured comfort-focused pathway rather than a standalone procedural option. If effective, SPING may represent a transition toward a targeted neuraxial strategy aimed at rapid analgesia, reduced opioid exposure, early upright positioning, and timely discharge aligned with palliative goals of care.

Nevertheless, several limitations warrant consideration. Firstly, the observational cohort design precludes random assignment, making it vulnerable to unmeasured confounding factors. Patients choosing SPING block may differ systematically from those receiving alternative treatment. To address potential confounding, we will perform detailed baseline assessments, including measures of frailty, comorbidity burden, and functional status, and incorporate these variables into multivariable regression models. Secondly, high early mortality and potential loss to follow-up in this frail population may limit the interpretability of long-term outcomes and distort Kaplan–Meier survival estimates. We will report censoring patterns transparently and perform sensitivity analyses to assess robustness. Thirdly, reliance on proxy-reported pain and QoL instruments introduces subjectivity and risk of misclassification. To reduce bias, we use validated tools (PACSLAC-D, EQ-5D-5 L proxy versions). Furthermore, while qualitative thematic analysis is invaluable for understanding experiences and implementation barriers and facilitators, it is inherently interpretative. Investigator triangulation, reflexive memoing, and audit trails will safeguard analytical rigour. Reflexive memoing involves making brief, continuous notes throughout the research process to document the researcher’s own thoughts, assumptions, and potential biases, thereby increasing transparency and awareness of how these may influence the analysis [30].

An additional consideration is the ethical and clinical complexity of decision-making in this setting. Hip fracture care is traditionally framed as a surgical emergency, even when surgery offers no meaningful benefit. This may create a default tendency toward operative management even in patients with limited potential for meaningful recovery [5, 22]. In frail older adults approaching the end of life, this may conflict with priorities centered on comfort, dignity, and minimising treatment burden. The implementation of SPING should therefore be understood not merely as the adoption of a technique, but as part of a structured, value-concordant non-operative pathway grounded in shared decision-making.

Heterogeneity in supportive care protocols (such as differences in analgesia and discharge criteria) across participating centres, which may influence secondary outcomes such as discharge timing and resource use. Local practice variation will be captured, and centre effects accounted for in mixed-effects models. Missing data, especially among the most vulnerable patients, may also arise. Multiple imputation will address this issue where appropriate. Despite these limitations, PALLIHIP’s pragmatic design mirrors real-world practice, enhancing generalizability. The integrated health economic component will further inform policymakers regarding the potential budgetary implications of broader SPING block adoption.

Taken together, PALLIHIP evaluates SPING block not only as a procedural innovation but as a potential cornerstone of a structured palliative non-operative hip fracture pathway. By generating prospective data on patient-centered outcomes, costs, and implementation, the study aims to reduce unwarranted variation in care and support the development of evidence-informed national guidance.

In conclusion, PALLIHIP will provide prospective insights into the effects of SPING block on pain, QoL, and end-of-life care in frail, older adults with a non-operatively managed hip fracture. By incorporating the perspectives of patients, informal caregivers, and professionals, the study seeks to support consistent, patient-centred, and goal-value concordant care in this vulnerable population.

## Data Availability

All data generated or analysed during the current study are available from the corresponding author upon reasonable request after study completion.
